# Recent Trends in Metal Nanoparticles Decorated 2D Materials for Electrochemical Biomarker Detection

**DOI:** 10.3390/bios13010091

**Published:** 2023-01-05

**Authors:** Aneesh Koyappayil, Ajay Kumar Yagati, Min-Ho Lee

**Affiliations:** School of Integrative Engineering, Chung-Ang University, 84 Heuseok-ro, Dongjak-Gu, Seoul 06974, Republic of Korea

**Keywords:** metal nanoparticles, immunosensor, MXene, MoS_2_, graphene, MOF, biomarkers, graphitic carbon nitride, black phosphorous, 2D-LDHs, boron nitrides, graphdiyne

## Abstract

Technological advancements in the healthcare sector have pushed for improved sensors and devices for disease diagnosis and treatment. Recently, with the discovery of numerous biomarkers for various specific physiological conditions, early disease screening has become a possibility. Biomarkers are the body’s early warning systems, which are indicators of a biological state that provides a standardized and precise way of evaluating the progression of disease or infection. Owing to the extremely low concentrations of various biomarkers in bodily fluids, signal amplification strategies have become crucial for the detection of biomarkers. Metal nanoparticles are commonly applied on 2D platforms to anchor antibodies and enhance the signals for electrochemical biomarker detection. In this context, this review will discuss the recent trends and advances in metal nanoparticle decorated 2D materials for electrochemical biomarker detection. The prospects, advantages, and limitations of this strategy also will be discussed in the concluding section of this review.

## 1. Introduction

The definition of biomarkers has evolved over time, and a broader definition was suggested by the World Health Organization as “a biomarker is any substance, structure, or process that can be measured in the body or its products and influence or predict the incidence of outcome or disease” [1,2]. More specific definitions such as “a biological molecule found in blood, other body fluids, or tissues that is a sign of a normal or abnormal process, or of a condition or disease and can be tested to see how well the body responds to treatment for a disease or condition” [3], and “a characteristic that can be objectively measured and quantitatively evaluated as an indicator of a normal biological and pathological process, or pharmacological responses to a therapeutic intervention” [4] were coined by the US National Cancer Institute, and the US National Institutes of Health, respectively. Biomarkers can be biological, chemical, or physical, and are measurable parameters indicative of a specific biological state. The detection of biomarkers is crucial for the diagnosis and treatment of numerous diseases [5]. Biomarkers are classified broadly into imaging biomarkers and molecular biomarkers based on their characteristics. Imaging biomarkers are often used in combination with various imaging tools, whereas molecular biomarkers comprise RNA, DNA, and proteins [6]. Molecular biomarkers are easily quantifiable from biological samples and can complement clinical characteristics [7,8]. Another category, known as pharmacodynamic biomarkers, is applied in drug development during dose optimization studies [9]. Based on the application, biomarkers are classified into prognostic biomarkers, diagnostic biomarkers, predictive biomarkers, and monitoring biomarkers [10]. Prognostic biomarkers help to identify the risk of disease progression in the future [11]. Diagnostic biomarkers help physicians to identify a specific disease condition [12], and predictive biomarkers predict the responses related to therapeutic interventions [11], whereas a monitoring biomarker is usually measured for assessing the status of a medical condition or disease [13].

An ideal biomarker sensor must capture the biomarker selectively from the complex biological matrix of interfering molecules. Although nonspecific binding is still a concern, electrochemical detection methods, specifically electrochemical impedance spectroscopy (EIS), allow the selective analysis of biomarker detections by the resistive and/or capacitive changes due to physical and/or biomolecular interactions of the electrode surfaces coated with nanomaterials, DNA, proteins, etc. [14,15,16]. It is one of the basic and widely used approaches to determine the fundamental redox events at the electrode-electrolyte interface. However, evaluations are made by comparing the results of the EIS with cyclic voltammetry (CV) measurements. Also, differential pulse voltammetry (DPV) and square wave voltammetry (SWV) techniques are used in biomarker detection systems for both label and label-free approaches [17,18]. Among these techniques, CV-based detection sensing is widely reported due to its ability to explain the electrochemical events, such as oxidation-reduction reactions and electron-transfer kinetics occurring at the electrode-electrolyte interface, and the mass transport towards the electrode surface [19,20,21]. The search for advanced functional materials for electrochemical biomarker detection has sparked a research interest in layered 2D materials over the past few years and several novel approaches were reported for the synthesis of various 2D materials and their nanocomposites with exciting immunosensor applications. The interest and demand for 2D materials have increased significantly, and the global market for 2D materials is expected to grow rapidly with a CAGR of 3.9% between 2020 and 2027 and a corresponding increase in valuation from 2.27 billion to 2.86 billion USD [22]. In this context, this review discusses the recent advances and challenges of metal nanoparticle decorated 2D materials for biomarker detection.

## 2. Metal Nanoparticles on 2D Materials for Biomarker Detection

Nanoparticles used separately or in conjugation with other nanomaterials on 2D materials fulfill various roles in the design and development of electrochemical immunosensors. Also, they improve the analytical characteristics of the developed sensors such as linear range, LOD, and sensitivity [23]. For instance, nanoparticles deposited on the surface of the working electrode result in an enhancement of the surface area, thereby leading to an increased molecule loading capacity [24,25]. Additionally, the unique properties of nanoparticles could enhance the signal for the sensitive determination of biomarkers [23]. Also, the high electrical conductivity of metal nanoparticles at the electrode surface accelerates the redox electron transfer process. In some cases, nanoparticles could act as platforms for anchoring antibodies [26]. Metal nanoparticles were also used as a transport medium to capture the analyte from the sample, thereby concentrating the analyte molecules towards the electrode surface to improve the analytical signal [27]. Among various metal nanoparticles, AuNPs were extensively used to immobilize antibodies on the electrode surface to effectively amplify the immunosensor signal, anchor antibodies, and improve electrocatalytic activity [28,29].

### 2.1. Graphene Oxide Conjugated with Nanoparticles for Electrochemical Biomarker Detection

**Figure 1:**
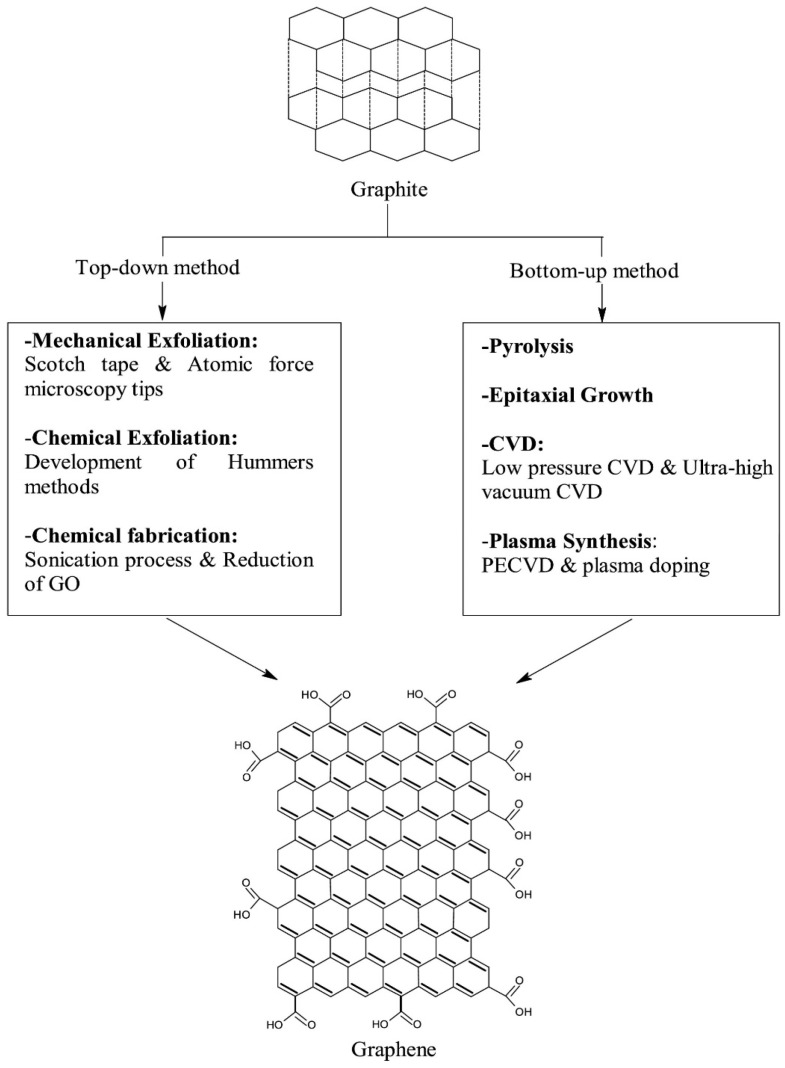
The schematic diagram for the synthesis of graphene. Reprinted with permission from Ref. [30]. Copyright 2018, Elsevier.

Graphene, a single layer (monolayer) of SP^2^ carbon atoms with a molecular bond length of 0.142 nm, is tightly bound in a hexagonal honeycomb lattice. It is basically extracted from graphite and is merely a sheet of graphite. Graphene possesses excellent electrical conductivity (200,000 cm^2^/Vs) due to its bonding and antibonding of pi orbitals, with the strongest compound around 100–130 times stronger than steel with a tensile strength of 130 GPa and a Young’s Modulus of 1 TPa-150,000,000 psi. It is also one of the best conductors of heat at room temperature (at (4.84 × 10^3^–5.30 × 10^3^ W/mK). As graphene is a subunit of graphite it can be synthesized by direct extraction from bulk graphite. From the high-quality sample of graphite, graphene can be extracted by micromechanical cleavage or the scotch tape method of production. It is a straightforward method that doesn’t need any specialized equipment. A piece of adhesive tape is placed onto and then peeled off the surface of a sample of graphite, resulting in a single to few layers of graphene. Other methods include the dispersion of graphite, exfoliation of graphite oxide, epitaxial growth, and chemical vapor deposition (CVD) as shown in Figure 1.

Graphene oxide is a form of graphene that includes oxygen functional groups and possesses interesting properties that are different from graphene. By reducing graphene oxide, these functional groups can be removed resulting in reduced graphene oxide. The production of reduced graphene oxide can be done in (i) chemical reduction, (ii) Thermal reduction; (iii) microwave and photoreduction; (iv) photocatalyst reduction; (v) solvothermal/hydrothermal reduction. The detailed information for various synthesis routes can be found elsewhere [31,32,33] and is beyond the scope of this review.

In this section, we discuss the development of various types of electrochemical sensors based on graphene oxide conjugated with nanoparticles that have been reported recently for various types of biomarkers. The development of biosensors that accurately measure the desired biomarker at high sensitivity and selectivity is crucial. However, sensitivity and selectivity are the two main factors that limit accuracy when performing the detections at the point of care with meager volumes of biological test solutions. For cancer cell analysis, the sensors should be able to detect tumors within the range of 100–1000 cell counts. To overcome these difficulties, innovative biosensor approaches with the optical, electrochemical, and piezoelectric transducer occupy the place of benchtop protocols adopted by the classical detection methods. Among these biosensors, electrochemical-based approaches competed with optical sensors which are widely used for the analysis of cancer biomarkers due to the characteristics of high sensitivity, selectivity, fast response, ease of use, low cost, and minimal fabrication procedures. In electrochemical biosensors, the right choice of transducer material is crucial, since it is the transducer that mainly influences the overall sensitivity [34] with minimal contributions from labeling methods.

**Figure 2:**
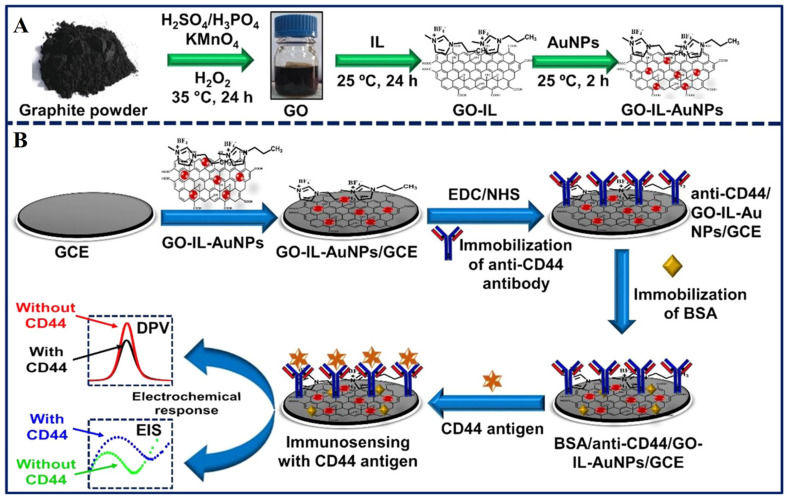
(**A**) Schematic diagram shows the synthesis of GO-IL-AuNPs hybrid nanocomposite and (**B**) Stepwise fabrication shows the surface modification procedures for the fabrication of BSA/anti-CD44/GO-IL-AuNPs/GCE Immunosensor. Reprinted with permission from Ref. [35] Copyright 2022, ACS.

Recently, Ranjan et al. [35] reported on the detection of breast cancer CD44 biomarkers using a gold-graphene oxide nanocomposite with ionic liquid with differential pulse voltammetry and electrochemical impedance spectroscopy. In this work, the authors reported the synthesis of RGO, ionic liquid (IL), and Au nanoparticles (Au NPs) by the citrate reduction method and other chemical procedures to form a nanocomposite on a glassy carbon electrode (GCE), as shown in Figure 2. In this work, the addition of 1-butyl-3-methylimidazolium tetrafluoroborate, an ionic liquid in conjugation with Au nanoparticles enabled the enhancement in the overall sensitivity of the developed sensor. Once the nanocomposite is deposited on GCE, the surface is activated with EDC/NHS to covalently bind the anti-CD44 antibodies. After the surface is blocked with BSA for nonspecific binding, then different concentrations of CD44 antigen were allowed for electrochemical investigation with CV, DPV, and EIS. The sensor possessed a linear range of 5 fg/mL to 50 µg/mL with a LOD of 2.7 fg/mL and 2.0 fg/mL in serum and PBS samples, respectively. This sensor is a promising candidate for the onsite detection of CD44 in breast cancer patients.

**Figure 3:**
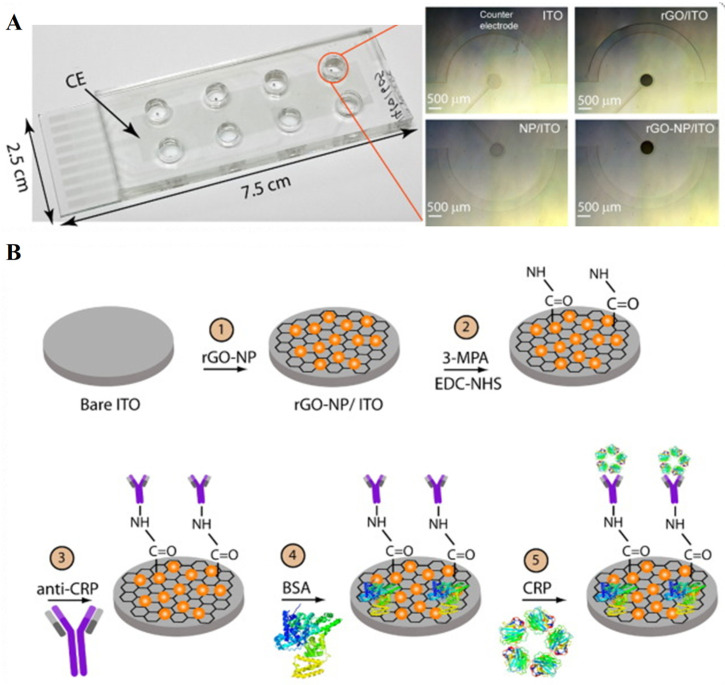
(**A**) Fabrication of 8-channel Indium-tin oxide electrodeposited with reduced graphene oxide-nanoparticle microdisk electrode array as working electrodes with a shared counter electrode. (**B**) Chemical functionalization of modified ITO electrode with EDC/NHS to couple antibodies for CRP detection in real samples. Reprinted with permission from Ref. [36]. Copyright 2016, Elsevier.

In another study, Yagati et al. [36] proposed indium tin oxide (ITO)-based electrodes modified with reduced graphene oxide-gold nanoparticles that were used for the electrochemical impedance sensing of the C-reactive protein in serum samples. This biomarker detection is crucial in analyzing the inflammation due to an infection, and the risk of heart disease. In this study, graphene oxide-Au nanoparticles were electrodeposited on ITO microdisk electrodes fabricated using standard photolithography techniques. Subsequently, the modified electrodes were coated with a self-assembled monolayer of 3-MPA and activated with EDC/NHS. After the surface-blocking protocol was performed, then the selective antibodies were immobilized on the rGO-NP surface. Once the transducer surface is ready, a different concentration of CRP in human serum (1: 200) was detected with the help of impedance spectroscopy (Figure 3). The key feature of this sensor is that by forming the nanohybrid materials (RGO-NP hybrid) on the electrode, it results in an enhanced sensitivity toward CRP detection. The linear range of the sensor is 1–1000 ng/mL with an LOD of 0.08 ng/mL in serum samples. Based on the findings, it has the feasibility to employ multiplexed assay detection of biomarkers for point-of-care applications.

**Figure 4:**
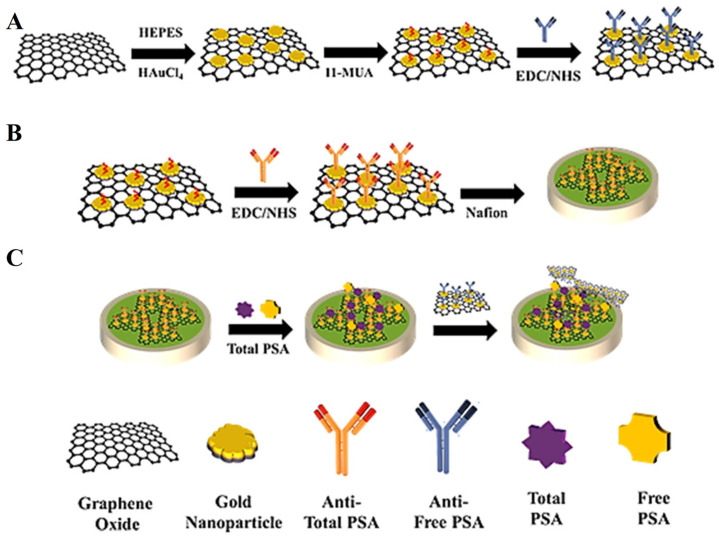
(**A**) Procedures for the fabrication of Go/GNP/Ab. (**B**) Procedure for preparing the electrochemical sensor. (**C**) Schematic illustration of the novel electrochemical sensor for PSA marker detection. Reprinted with permission from Ref. [37]. Copyright 2019, Wiley.

Jonous et al. [37] reported on the detection of prostate-specific antigen (PSA) by using a sandwich-type transducer composed of graphene oxide (GO) and gold nanoparticles (AuNPs). In this work the authors utilized an 11-mercaptoundecanoic acid for self-assembled monolayer formation on the GO-coated glassy carbon electrode (GCE) and a subsequent modification with EDC/NHS to convert -COOH to -NH for antibody bindings (Figure 4). After blocking with 1% BSA, different concentrations of PSA were allowed to bind to the electrode and with square wave voltammetry, and the quantification was made. The sensor possessed a limit of detection estimated to be around 0.2 and 0.07 ng/mL for total and free PSA antigens, respectively. The incorporation of AuNPs on GO/GCE enabled double functionality, i.e., specific recognition and signal amplification, for sensitive determination of PSA.

**Figure 5:**
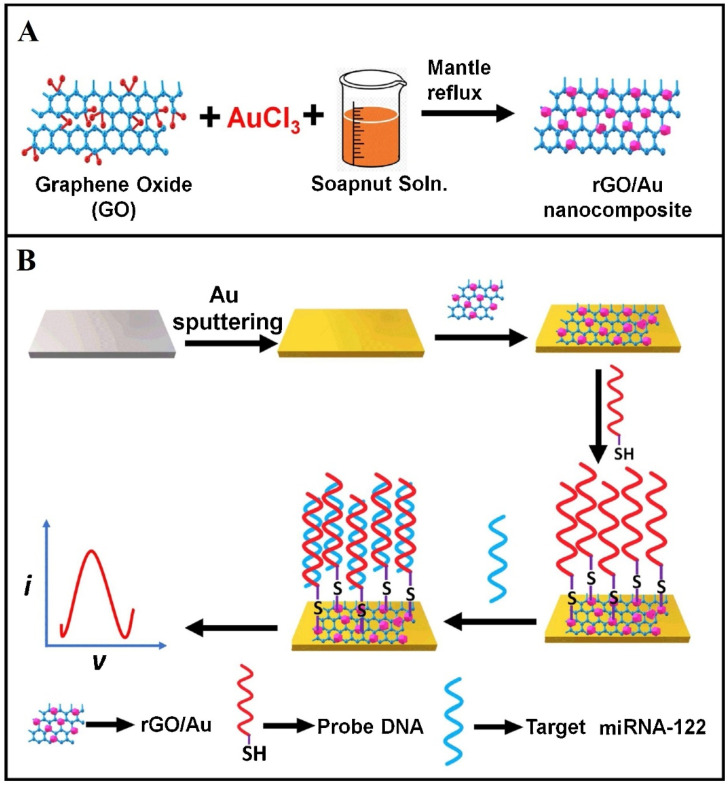
Schematic representation of the (**A**) Synthesis of rGO/Au nanocomposite, (**B**) Fabrication of rGO/Au nanocomposite-based miRNA-122 electrochemical detection platform. Reprinted with permission from Ref. [38]. Copyright 2021, Elsevier.

Also, Kasturi et al. [38] reported on the development of a biosensor for the detection of microRNA-122 (miRNA-122) with AuNPs-decorated reduced graphene oxide (rGO) on the Au electrode surface (Figure 5). The thiol-labeled DNA probes were attached to the Au-rGO transducer surface by forming a SAM layer, with subsequent blocking with 1% BSA. Then, the target miRNA was allowed to bind to the transducer surface to quantify the biomarker for liver diseases.

The sensor possessed a linear range from 10 µM to 10 pM and had a detection limit of 1.73 pM. The sensor possessed good biocompatibility, superior electron transfer characteristics, large surface area, and selective conjugation with biomarkers. Also, the sensor design can be applied to construct other types of biomarker detection. Furthermore, it can be integrated with a lab on a chip platform. It is also applicable to the large-scale production of sensors with a focus on the early detection of diseases.

**Figure 6:**
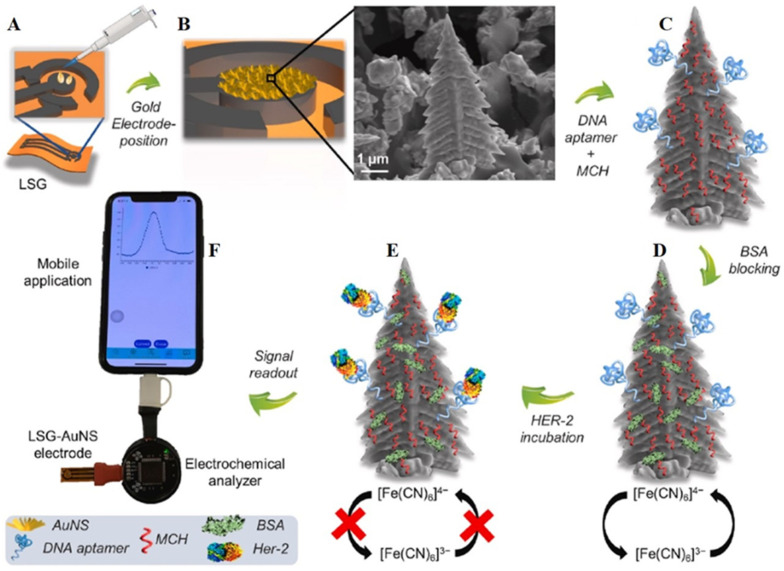
The schematic diagram for the formation of laser-induced graphene (LIG) electrode sensor. (**A**) LIG electrode on polyimide sheet, (**B**) Formation of Au nanostructures on working electrode area with electrodeposition, inset shows the SEM images of the tree-like structure of Au. (**C**) Bindings of DNA aptamer on the electrode through self-assembly of mecaptohexanol (MCH), (**D**) Surface blocking procedures with BSA and measurement of electrochemical signal with [Fe(CN)_6_]^3−/4−^ redox probe, (**E**) Incubation with the HER-2 antigen and measurement of EC signal, and (**F**) Quantification of HER-2 by evaluating the electrochemical signal. Reprinted with permission from Ref. [39]. Copyright 2021, Elsevier.

In another interesting work, Rauf et al. [39] reported on the use of laser-induced graphene oxide [34] as a new-generation electrode in cancer research for the detection of human epidermal growth factor receptor 2 (HER-2). In this study, with laser printing technology, the structures of working, counter, and reference electrodes were formed on a polyimide sheet, then the gold nanostructures (Christmas-tree-like structures) were formed by electrodeposition on the working electrode (Figure 6). Subsequently, the sensor surface is modified with thiol labeled HER-2 aptamer and blocked with BSA for any nonspecific bindings. Then, the HER-2 protein was allowed, in different concentrations, to interact with the aptamer immobilized surface. The electrochemical signals were then recorded for the aptamer surface after bindings with different concentrations with [Fe(CN)_6_]^3−/4−^ redox probe. The CV analysis showed a decrease in current upon bindings of various concentrations of HER-2, and from the calibration, the limit of detection was found to be 0.008 ng/mL. It is claimed that with the incorporation of 3D Au nanostructures the sensor possessed a high electron transfer rate, which resulted in achieving a lower LOD and possessing high sensitivity and accuracy in detecting HER-2 in human serum samples. Furthermore, special software was developed to make it a POC device, in which the laboratory aptasensor could be converted into a hand-held aptasensor.

**Figure 7:**
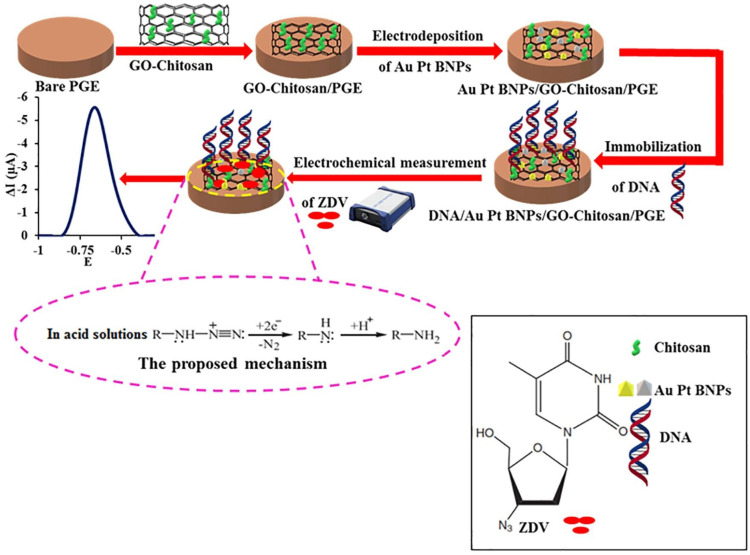
Schematic route for the fabrication of DNA/Au−Pt BNPs/GO−chit/PGE transducer surface for the development of an electrochemical biosensor for the detection of ZDV. Reprinted with permission from Ref. [40]. Copyright 2021, Elsevier.

Also, Hasanjani et al. [40] reported on the development of Zidovudine (ZDV). A modified pencil graphite electrode (PGE) was made using deoxyribonucleic acid/Au-Pt bimetallic nanoparticles/graphene oxide-chitosan (DNA/Au-Pt BNPs/GO-chit/PGE) (Figure 7). The PGE was immersed in the GO-chit solution to create the graphene oxide-chitosan/pencil graphite electrode (GO-chit/PGE). Later, the electrodeposition of Au-Pt bimetallic nanoparticles (Au-Pt BNPs) was accomplished on the surface of the GO-chit/PGE-modified electrode. Subsequently, DNA was immobilized on the Au-Pt BNPs/GO-chit/PGE, applying a constant potential of 0.5 V. 

Using differential pulse voltammetry, the I−V response was recorded for different concentrations of ZDV. The sensor showed a linear dynamic range from 0.01 pM to 10.0 nM, with a detection limit of 0.003 pM in human serum samples. 

Recently, Kangavalli and Veerapandian reported on the development of a dengue biomarker using ruthenium bipyridine complex on the surface of graphene oxide [41]. They also reported on various EC-based techniques for the electrodeposition and electroless deposition procedures of graphene oxide as a nanoarchitecture for a label-free biosensor platform [42]. Some more information on electrochemical biosensors developed for biomarker detection that contain graphene oxide and metal nanoparticles can be found in some valuable studies recently reported, and are available in the literature [43,44,45,46,47]. Graphene oxide-based nanomaterials offer a wide range of possibilities for developing sensitive electrochemical biosensors for biomarker detection. In recent years, significant advances in graphene-nanoparticle-based electrochemical sensors are made for the detection of cancer biomarkers, and here we analyze the analytical parameters of those sensors, as shown in Table 1.

### 2.2. MoS_2_ Conjugated Nanoparticles for Electrochemical Biomarker Detection

**Figure 8:**
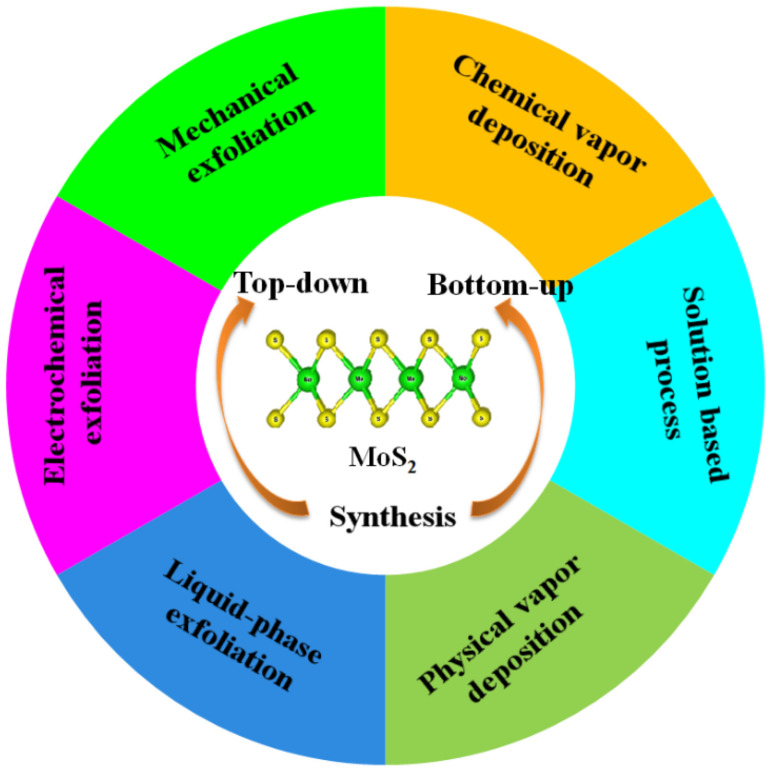
Various synthetic methods for MoS_2_ preparation. Reprinted with permission for Ref. [74]. Copyright 2022 MDPI.

Recently, transition metal dichalcogenides (TMDCs) found their applications in various biosensors due to their large surface-to-volume ratio, tunable electronic and optical properties, low toxicity, and unique van der Waals layered structure [65]. In TMDCs, one layer of transition metal atoms (M) lies between two layers of chalcogen atoms (X) resulting in a formula MX_2_. Various kinds of TMDCs can be realized by altering the chalcogen atoms such as Sulphur (S), Selenium (Se), and Tellurium (Te), and metal atoms like Molybdenum (Mo) and Tungsten (W). Among these, MoS_2_ is commonly used because its fundamental constituents are surplus and innoxious [66]. MoS_2_ molybdenum (Mo) atoms lie between the two sulfide atoms layers (S-Mo-S) and atoms in the crystal are associated by strong covalent bonding and adjacent layers of MoS_2_ are held by weak van der Waals forces. MoS_2_ possesses a mobility of 200 cm^2^/Vs at room temperature, high on/off current ratio of 10^8^, and a direct band gap of 1.8 eV. Based on these properties, MoS_2_ becomes a promising alternative to graphene and is applied in various electrochemical and optical sensors [67,68,69]. MoS_2_ can be synthesized in both top-down and bottom-up approaches (Figure 8). The top-down approach includes the exfoliation of MoS_2_ [70], while the bottom-up approaches include (i) chemical vapor deposition [71]; (ii) physical vapor deposition [72]; (iii) solution-based processing [73]. For a more detailed synthesis of MoS_2_, readers are encouraged to go through the literature survey of the desired synthesis approach. Thus, like graphene, MoS_2_ offers a large surface area that enhances its biosensing performance. 

**Figure 9:**
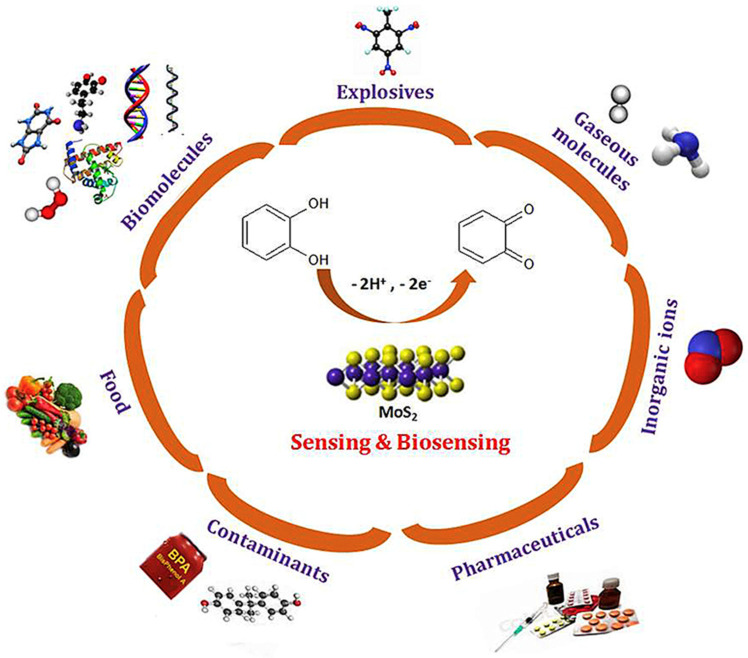
MoS_2_ nanostructures-based electrochemical sensing application in various fields. Reprinted with permission from Ref. [76]. Copyright 2018, Elsevier.

MoS_2_ possesses a direct band gap of 1.8 eV in the monolayer, lattice defects of zero dimensionality, grain boundary defects, and an enhanced surface-to-volume ratio. Also, the feasibility of surface modification and chemical functionalization makes these characteristics of MoS_2_ to adopt and study in scientific and industrial fields [75] (Figure 9). Furthermore, to increase the electroactivity/conductivity of graphene and/or other 2D materials, mostly nanoparticles were incorporated to achieve the synergistic effects from both nanomaterials, which ultimately resulted in an improvement in the overall analytical performance of the biosensor. In this section, we review various types of biosensors that incorporate metal nanoparticles on MoS_2_ for the detection of various biomarkers.

**Figure 10:**
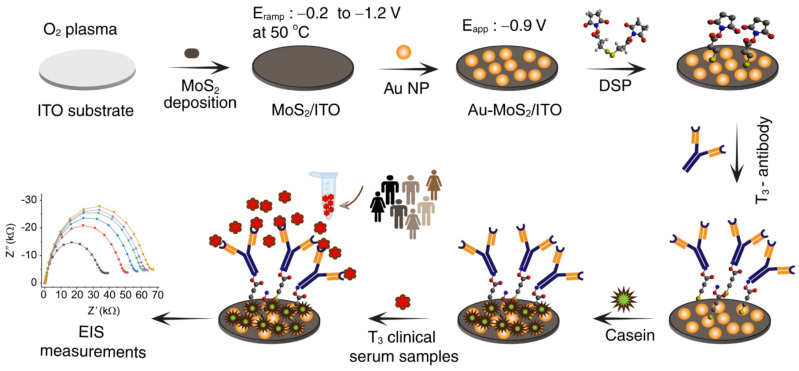
Schematic illustration of the total triiodothyronine (T_3_) receptive interface fabrication through the immobilization of the antibody on a step-by-step modification process of MoS_2_–Au formation and subsequent functionalization with a dithiobis (succinimidyl propionate) monolayer on an indium tin oxide electrode surface. With increasing concentration of the T3 analyte in serum, the EIS (Nyquist plot) shows increased semi-circle (Rct) for quantification. Reprinted with permission from Ref. [77]. Copyright 2020, Elsevier.

In a recent report that mentions the usage of MoS_2_-Au nanoparticles, Yagati et al. [77] reported on the applications of MoS_2_ conjugated Au nanoparticles on indium tin oxide (ITO) electrodes for the detection of the thyroid-stimulating hormone biomarker, triiodothyronine (T_3_), as shown in Figure 10. Electrodeposition procedures allowed the formation of MoS_2_ and Au nanostructures on the ITO electrode. Subsequently, T_3_ antibodies were immobilized on the MoS_2_-Au/ITO surface by forming a self-assembled monolayer of dithiobis (succinimidyl propionate) (DSP). For any nonspecific bindings, the surface is coated with casein and then subjected to different concentrations of the T_3_ biomarker diluted in both PBS and serum samples. Electrochemical impedance spectroscopy was used to analyze the bindings of T_3_ to its antibodies and a linear correlation was observed for different concentrations. Based on the quantifications made by this sensor for the detection of T_3_, a linear range of 0.01–100 ng/mL with a detection limit of 2.5 pg/mL was observed. The sensor also showed a good correlation with data observed by the conventional method (Roche Cobas) and possessed high sensitivity and selectivity in discriminating the healthy and cancer samples. Based on the findings, the developed sensor could apply to cancer-related biomolecule analysis.

**Figure 11:**
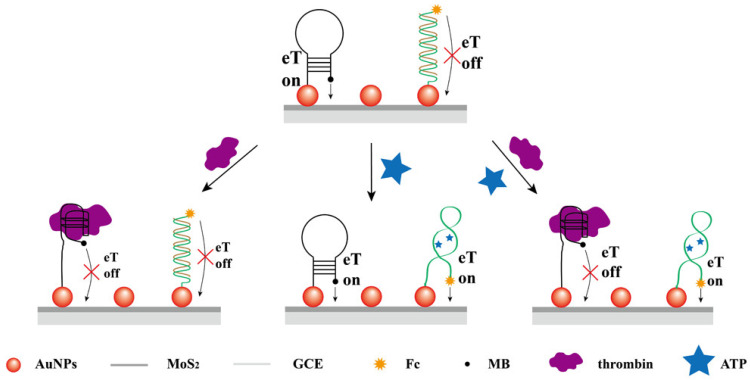
Schematic representation for the development of the aptasensor for the determination of ATP and thrombin. Reprinted with permission from Ref. [78]. Copyright 2016, ACS.

Su et al. [78] developed dual target sensing (adenosine triphosphate (ATP) and thrombin) detection electrochemical biosensors based on gold nanoparticles-decorated MoS_2_ (AuNPs–MoS_2_) nanocomposites which feature both “signal-on” and “signal-off” elements in the detection system, and thrombin and ATP could act as inputs to activate an AND logic gate (Figure 11). In this approach, two different aptamer probes labeled with redox tags (ferrocene (Fc) and methylene blue (MB)) were simultaneously immobilized on an AuNPs-MoS_2_ modified glassy carbon electrode (GCE) through Au-S bond formations. Subsequently, the electrode was immersed in 6-mercaptohexanol to block the uncovered spots of AuNPs–MoS_2_/GCE. Square wave voltammetry (SWV) was used to determine the various concentrations of ATP and thrombin applied to the GCE. From concentration vs. change in the current results, it was evaluated that the sensor had a linear range for the determination of ATP, which was 1 nM to 10 mM with a detection limit of 0.32 nM, while for the thrombin determination, the linear range was 0.01 nM to 10 µM with a detection limit of 0.0014 nM. 

**Figure 12:**
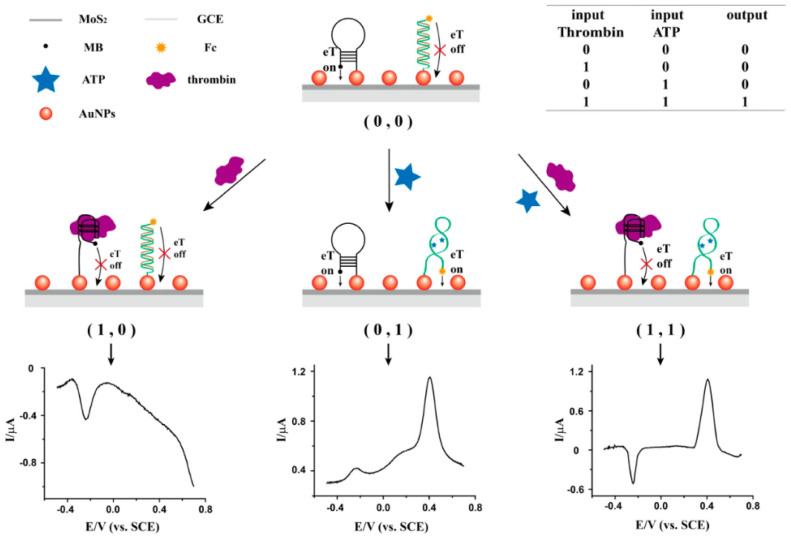
Schematic description of the MoS_2_-Based AND logic gate for determination of ATP and thrombin. Reprinted with permission from Ref. [78]. Copyright 2016, ACS.

The authors also suggested that this mechanism can be acted as an AND logic gate by using ATP and thrombin as inputs and the electrochemical signals of Fc and MB as outputs (Figure 12). The logic gate works on the structural conversion of the aptamer probe triggered by ATP and thrombin. The working mechanism was the individual peak current enhancement of Fc or the suppression of MB as electron transfer OFF (eTOFF) or “zero” output, and the simultaneous peak current enhancement of Fc and suppression of MB as electron transfer ON (ON) or “one” output. From the inset table, a “one” output was achieved only when both inputs were “one”. When there were no inputs (0, 0) or only one input (0, 1 or 1, 0), the result was “zero” output. Thus, the MoS_2_-based multiplexed aptasensor could also serve as an “AND” gate.

**Figure 13:**
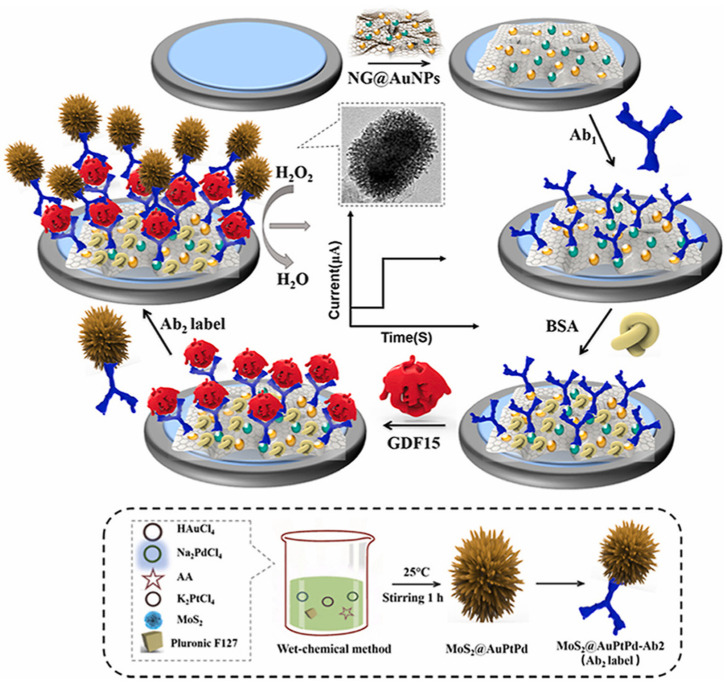
Schematic illustration for the development of a sandwich-type electrochemical sensor for GDF-15 detection sensor. Reprinted with permission from Ref. [79]. Copyright 2022, Elsevier.

In another work, Chen et al. [79] reported on the development of a growth differentiation factor-15 (GDF-15) expression sensor which is a potential biomarker for the diagnosis, risk stratification, and prognosis of various cardiovascular diseases (Figure 13). Here, a sandwich-type immunosensor was constructed using amine-modified graphene-supported gold nanorods (NG/AuNPs) as a substrate platform, and the durian-shaped MoS_2_/AuPtPd nanodendrite (NDs) as a label for secondary antibodies (Ab_2_) for the quantification of growth differentiation factor-15 (GDF-15). NG/AuNPs are used to enhance the surface area and for the immobilization of primary antibodies through the binding of amino or sulfhydryl groups. Subsequently, the electrodes were blocked with 1wt% BSA. Finally, the signal probe MoS_2_/AuPtPd-Ab_2_ was added to the sample. 

The developed sensor was also applied to evaluate the efficacy towards the clinical sample analysis and compared with traditional sensing methods, such as ELISA, to evaluate the accuracy of the results. The sensor showed a linear range of 1.5 pg/mL to 1.5 µg/mL with a detection limit of 0.9 pg/mL. Due to its high sensitivity, rapid response, and feasibility to miniaturization, the proposed sensor could be applied to a point-of-care diagnostic tool for cardiovascular diseases and paves the path toward “liquid biopsies”.

**Figure 14:**
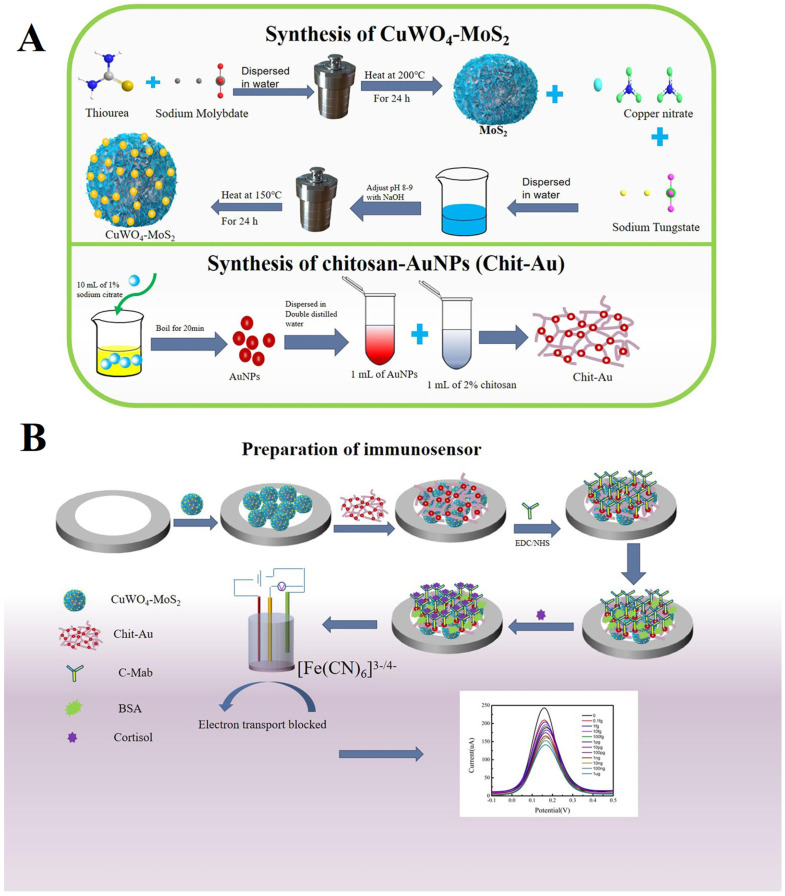
Schematic representation for (**A**) Synthesis of MoS_2_, CuWO_4_@MoS_2_, AuNPs, and Chit-Au nanocomposites; (**B**) Preparation process of the immune electrode. Reprinted with permission for Ref. [80]. Copyright 2022, Elsevier.

Nong et al. [80] reported on the detection of cortisol which is a glucocorticoid hormone that adrenal glands produce and release, and this hormone regulates stress, inflammation, blood pressure, sugar, and overall metabolism. In this work, copper tungstate-molybdenum sulfide (CuWO_4_@MoS_2_) and chitosan-gold (Chit-Au) nanocomposite were synthesized and applied to GCE (Figure 14). Subsequently, the cortisol antibody (C-Mab) was immobilized using the EDC/NHS reaction and subsequent blocking with BSA. Once the transducer surface was fabricated, SWV was performed to analyze the bindings of various concentrations of cortisol and a linear relationship was observed concerning different concentrations. The sensor showed a linear range of 0.1 fg/mL to 1 µg/mL with a detection limit of 0.014 fg/mL (S/N = 3). The sensor showed excellent storage stability and reproducibility and it can detect the content of cortisol in saliva.

**Figure 15:**
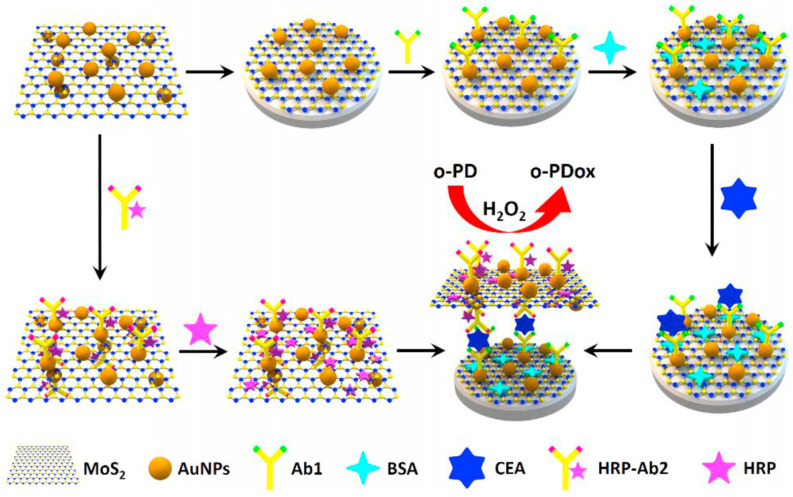
The schematic diagram for the stepwise modification of the GCE with MoS_2_ and Au nanoparticles for anti-CEA antibody immobilization for developing a CEA detection sensor. Reprinted with permission from Ref. [81]. Copyright 2019, Elsevier.

Su et al. [81] reported on the use of a MoS_2_-Au nanocomposite for the detection of a carcinoembryonic antigen (CEA). In this work, CEA antibodies labeled with horseradish peroxidase resulted in an amplified electrochemical signal by catalyzing o-phenylenediamine (o-PD) in the presence of hydrogen peroxide (H_2_O_2_). As can be seen in Figure 15, the MoS_2_-Au conjugated HRP labeled antibodies enhance the overall sensitivity when the different concentrations of CEA were measured using cyclic voltammetry. From the analytical performance, the sensor displayed a linear range of 10 fg/mL to 1 ng/mL with a detection limit of 1.2 fg/mL. The sensor also exhibited good stability, and high selectivity suggesting that the proposed immunosensor could detect CEA in real samples.

**Figure 16:**
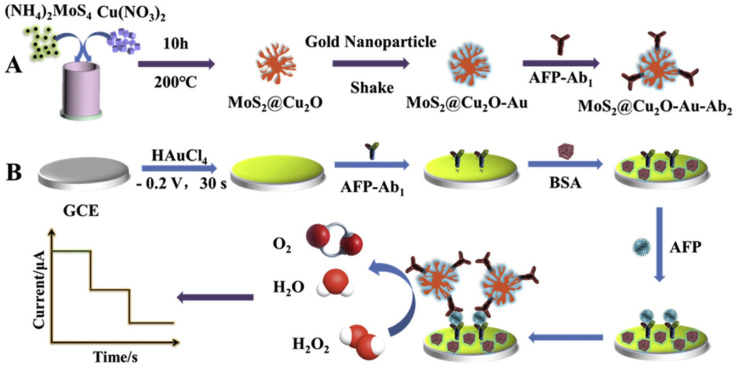
The schematic diagram for the preparation procedure for the sandwich-type electrochemical immunosensor. Reprinted with permission from Ref. [82]. Copyright 2019, Elsevier.

Also, Ma et al. [82] reported similar works using MoS_2_@Cu_2_O-Au nanoparticles for the detection of alpha-fetoprotein (AFP), a tumor marker to identify adult primary liver cancer (Figure 16). In this work, AuNPs were electrodeposited on GCE which acted as antibody carriers and sensing platforms. Further, MoS_2_@Cu_2_O was combined with the AuNPs as a strategy to obtain the signal amplification resulting in a composite MoS_2_-Cu_2_O-Au as a triamplification electrochemical signal. A sandwich immunosensor was developed by immobilizing primary antibodies on Au-deposited GCE and blocked with a surface with BSA for nonspecific bindings. Then, the electrodes were dipped with different concentrations of AFP. Subsequently, the HRP-labeled secondary antibodies coupled with MoS_2_@Cu_2_O were then allowed to conjugate with the electrode. Amperometric response, under suitable experimental conditions, exhibited that the sensor possessed a linear range of 0.1 pg/mL to 50 ng/mL and a detection limit of 0.037 pg/mL (S/N = 3). The sensor showed satisfactory recoveries when tested in human serum samples, and the proposed approach could extend the potential application of electrochemical immunosensors to medical applications.

Likewise, several reports demonstrated the usage of a MoS_2_-Au nanocomposite for the detection of electrochemical biosensors for various types of biomarker detection in clinical applications. However, very few reports show the possibility of point-of-care applications. Here, we analyzed the analytical parameters of the reports that adopt the MoS_2_-Au nanocomposite used for electrochemical sensors and presented them in the following Table 2.

### 2.3. Biomarker Detection on MXenes Conjugated with Metal Nanoparticles 

**Figure 17:**
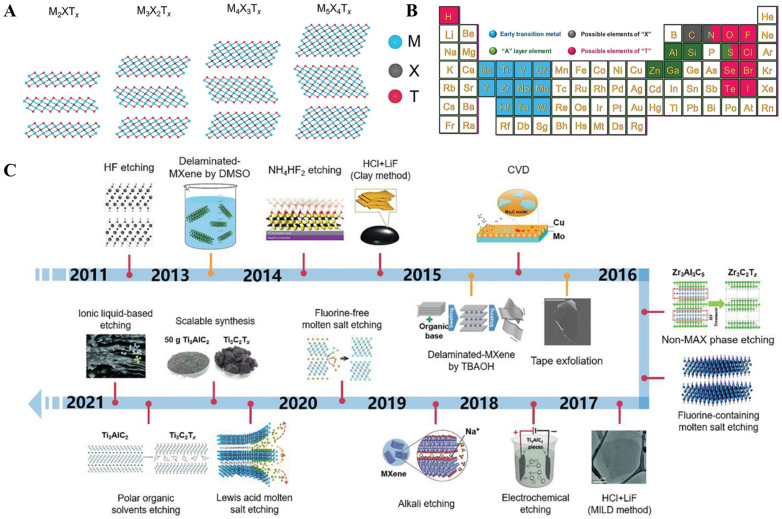
(**A**) Structure of various MXenes with surface terminations. (**B**) Periodic table elements experimentally used for the synthesis of MXenes, and (**C**) Timeline of the various synthesis routes to MXenes. Reproduced with permission from Ref. [117]. Copyright 2021, Wiley.

**Figure 18:**
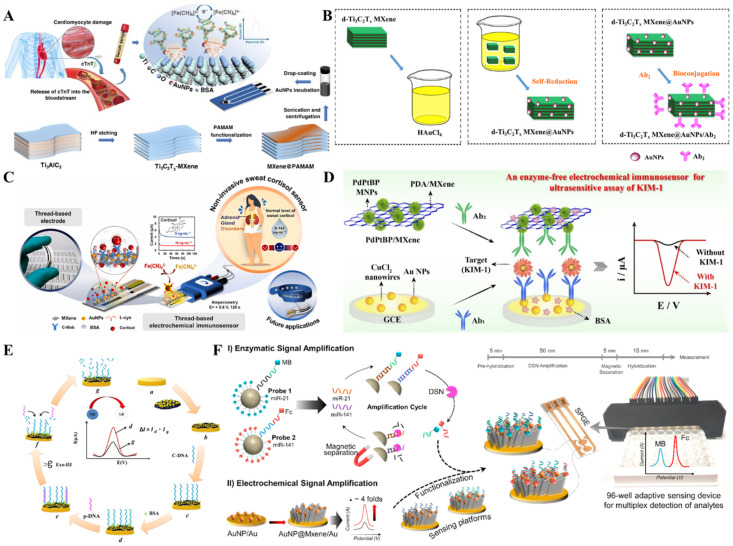
(**A**) Schematic illustration of the fabrication of AuNPs/MXene@PAMAM for the electrochemical detection of cTnT. Reproduced with permission from Ref. [108]. Copyright 2022, Nature. (**B**) Preparation of d-Ti_3_C_2_ MXene@AuNPs/Ab2 for the detection of PSA. Reproduced with permission from Ref. [109]. Copyright 2020, Elsevier. (**C**) Fabrication of L-cys/AuNPs/MXene on a thread-based electrochemical biosensor for noninvasive sweat cortisol detection. Reproduced with permission from Ref. [110]. Copyright 2022, Elsevier. (**D**) Fabrication of PdPtBP nanoparticles/MXene-based enzyme-free electrochemical biosensor for the detection of kidney injury molecule-1 (KIM-1). Reproduced with permission from Ref. [111]. Copyright 2021, Elsevier. (**E**) Schematics of the AuNPs-based cascaded signal amplification process for the detection of miRNA-21. Reproduced with permission from Ref. [113]. Copyright 2022, ECS, and (**F**) Schematic diagram based on AuNPs decorated MXene for the multiplex and concurrent detection of miR-21 and miR-141. Reproduced with permission from Ref. [114]. Copyright 2020, Elsevier.

MXenes are transition-metal carbides/nitrides/carbonitrides with a 2D structure and general formula M_n + 1_X_n_T_x_ (n = 1–3), where M is an early transition metal, X can be carbon or nitrogen, and T_x_ corresponds to the surface terminations (Figure 17A,B). The ideal electronic structure [95], structural stability [96], high surface-to-volume ratios [97], outstanding mechanical [98] and optical properties [99], versatile surface chemistries [100], tunable bandgap [101], and high thermal and chemical stability [102,103] make them promising materials for biomarker detection (Table 3). The initial synthesis approach for MXenes was realized based on the etching of Ti_3_AlC_2_ with 50% HF for 2 h at room temperature [104]. Later many environmentally friendly approaches were formulated [105] (Figure 17C). However, similar to any other pristine 2D materials, MXenes suffer from poor selectivity, low sensitivity, and slow response [106]. These disadvantages were usually overcome by synthesizing MXene-metal nanoparticle nanocomposites. MXene-metal nanoparticle nanocomposites possess a large specific surface area, superior electron conductivity, and enhanced electron transfer properties for biosensing applications [107]. To expand beyond the limitations of MXenes, Liu et al. [108] reported the covalent grafting of PAMAM onto MXene (MXene@PAMAM) (Figure 18A). Here, the PAMAM acted as an efficient stabilizer and spacer, thereby preventing the restacking and oxidation of the MXene. Moreover, the aminoterminals of PAMAM acted as adsorption sites for AuNPs. The AuNPs@MXene@PAMAM nanobiosensing platform was applied for the detection of the cardiovascular disease biomarker cTnT. The sensor performance was remarkable with a wide detection range (0.1–1000 ng/mL) and a very low detection limit (0.069 ng/mL). Medetalibeyoglu et al. [109] fabricated a d-Ti_3_C_2_T_X_ MXene@AuNPs/Ab2 bioconjugate-based sandwich-type electrochemical immunosensor for the detection of PSA. Here, AuNPs at the bioconjugate were used to label PSA secondary antibody-2 for signal amplification (Figure 18B). In one study, Laochai et al. [110] fabricated thread-based L-Cys/AuNPs/MXene working electrodes for the noninvasive electrochemical detection of sweat cortisol, which is an important biomarker for identifying adrenal gland disorders (Figure 18C). Here, MXene served as a 2D platform to anchor the monoclonal anticortisol antibodies, whereas AuNPs increased the specific surface area, and thereby the sensitivity of the detection system. Mesoporous nanoparticles (MNPs), comprising metallic and nonmetallic counterparts, show better catalytic performance compared to their bulk nanoparticles [111]. Liu et al. [112] reported sandwich-type PdPtBP MNPs/MXene-based immunosensor for the ultrasensitive detection of urine kidney injury molecule-1(KIM-1) (Figure 18D). Yang et al. [113] reported an interesting cascaded signal amplification strategy on in situ reduced gold nanoparticle deposited Ti_3_C_2_ MXene (Figure 18E), where MXene acted as a stabilizer and reductant. Here, AuNPs with the predominant (111) facet on MXene provided high electrocatalytic activity and were also used as a carrier of the C-DNA and to make DNA hybridization. Mohsen et al. [114] reported Au nanoparticles on Ti_3_C_2_ MXene for synergistic signal amplification (Figure 18F). Here, the perfectly distributed Au nanoparticles on the flaky architecture of MXene contributed to the enhanced electrochemical performance and the attomolar detection of multiple micro-RNAs (miRNAs) achieved on an AuNP@MXene/Au electrode. Wang et al. [115] proposed a competitive electrochemical aptasensor for the breast cancer biomarker Mucin1 based on Au nanoparticles decorated Ti_3_C_2_ MXene. Here, aptamer binding to the electrode surface was achieved through Au-S bonds by the electrodeposited gold nanoparticles. The electrochemical aptasensor reported a wide linear range (1.0 pM–10 μM) and a low detection limit (0.33 pM) with promising clinical applications. Cheng et al. [116] demonstrated a gold nanoparticle-modified MXene-based sandwich-type immunosensor platform for squamous cell lung cancer cytokeratin fragment antigen 21-1 (CYFRA 21-1). 

### 2.4. MOFs Conjugated Metal Nanoparticles for Electrochemical Biomarker Detection

**Figure 19:**
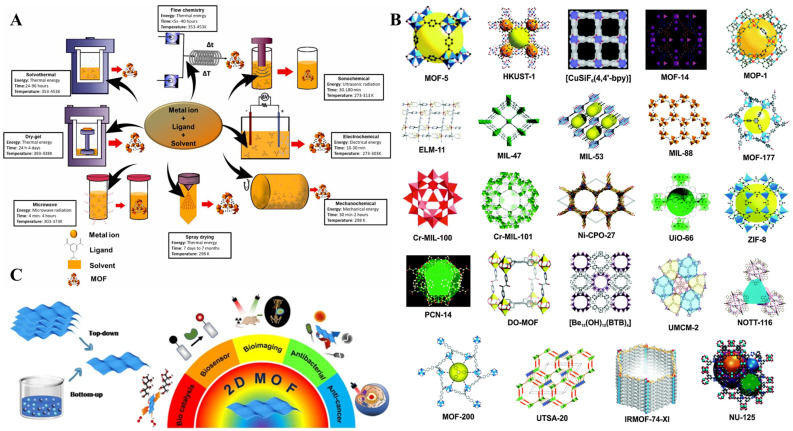
(**A**) Various literature reported conditions and approaches for the synthesis of MOFs. Reprinted with permission from Ref. [153]. Copyright 2021, Elsevier. (**B**) Structures of porous MOFs reported by several research groups. Reprinted with permission from Ref. [154]. Copyright 2015, Royal Society of Chemistry. (**C**) Various biomedical applications of 2D MOFs. Reprinted with permission from Ref. [155]. Copyright 2022, BMC (Springer).

**Figure 20:**
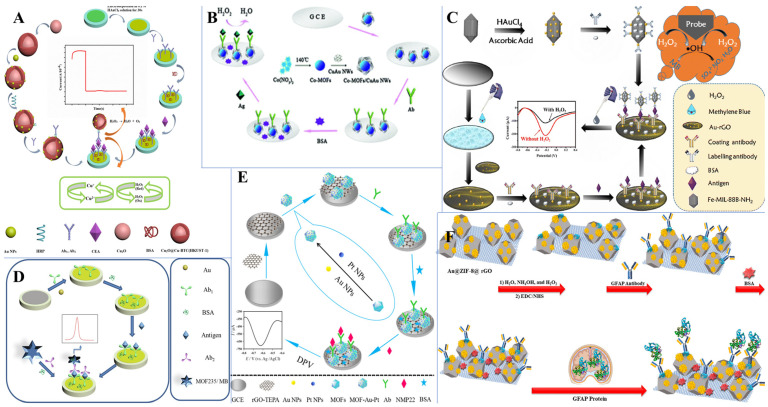
Schematic illustrations of (**A**) Fabrication of core-shell Cu_2_O@Cu-MOF@AuNPs-based electrochemical immunosensor for CEA detection. Reproduced with permission from Ref. [146]. Copyright 2020 Springer, (**B**) Preparation of Co-MOFs/CuAu NWs based label-free immunosensor for the detection of NMP-22. Reproduced with permission from Ref. [149]. Copyright 2019 Royal society of chemistry, (**C**) Fabrication of Au-MOF-based amperometric immunosensor for the detection of PSA. Reproduced with permission from Ref. [150]. Copyright 2020 Springer, (**D**) Preparation steps of AuNPs decorated MOF235/MB based electrochemical immunosensor for PSA detection. Reproduced with permission from Ref. [28]. Copyright 2021 Elsevier, (**E**) Stepwise assembly of AuNPs-PtNPs-MOFs based electrochemical immunosensor for the detection of NMP-22 in urine samples. Reproduced with permission from Ref. [152]. Copyright 2019 Elsevier, and (**F**) Preparation of GFAP-BSA-Anti-GFAP-Au@ZIF-8@rGO/SPE based electrochemical immunosensor for the detection of GFAP. Reproduced with permission from Ref. [151]. Copyright 2022 ACS.

As an emerging material with exceptional properties, metal-organic frameworks (MOFs) have been studied exceptionally during the past decades. MOFs are porous materials comprising a framework of metal ions or metal-containing clusters and organic ligands [122]. MOFs have been reported to have excellent properties such as a tunable structure [123], large surface area [124], abundant functional groups [125], high porosity [126], good conductivity [127], and thermal stability [128]. MOFs have been traditionally synthesized by hydrothermal/solvothermal methods [129]. The solvothermal method is a general concept where a solvent other than water is used, and the synthesis is usually performed at a temperature above the boiling temperature of the solvent in closed chemical reactors at higher pressures. Moreover, the greater pressure inside the closed reactor results in enhanced salt solubility. The benefits of the solvothermal process allowed researchers to develop reproducible protocols with total control of the long-term synthesis processes. The solvothermal method has the advantage of higher product yield with improved crystallinity [130]. The hydrothermal/solvothermal method has been optimized for the synthesis of MOFs such as Ni-MOF [131], Co-MOF [131], Fe-MOF [132], Cu-MOF [133], Zn-MOF [134], and mixed-ligand metal-organic frameworks [135]. In recent years, electrochemical synthesis gained attention, and several MOFs such as Cu_3_(HHTP)_2_ [136], Mn-DABDC(ES) [137], 2D/3D Zn(II)-MOF hybrid [138], Fe-MIL-101 and Fe-MIL-101-NH_2_ [139], etc. have been reported for various MOFs’ electrocatalytic applications. Electrochemical synthesis has the advantages of mild synthesis conditions, shorter synthesis times, and controllability of morphology and thickness by the applied current/voltage [140]. During electrochemical synthesis, the metal ions enter the solution through the dissolution of the anode and the process is usually continuous with the availability of dissolved linker molecules [141]. Researchers have also developed a variety of other synthesis approaches such as ultrasound and microwave-assisted [142], mechanochemical [143], and sonochemical [144] methods for the synthesis of MOFs with different morphology and applications (Figure 19). As shown in Table 4, modified MOF nanocomposites often outperform unmodified MOF and are often exploited for diverse biosensor applications [145]. MOFs are often decorated with metal nanoparticles in immunosensor applications for anchoring antibodies and enhancing the electrochemical signal. Nanoparticles decorated MOFs with versatile ligands and metal clusters, low cost, and simple operation provide researchers with an adequate 2D platform for biosensing applications. Li et al. [146] fabricated such an interesting immunosensor platform with core-shell Cu_2_O@Cu-MOF@AuNPs nanostructures for the sensitive detection of CEA (Figure 20A). Here, the sandwich-type electrochemical immunosensor achieved a tripled electrical signal amplification due to the synergistic effect of Cu-MOF, Cu_2_O, and AuNPs. Nanowires had more surface area to accommodate proteins and were used to fabricate label-free sensors with exceptional performance [147,148]. Li et al. [149] constructed such an ultrasensitive label-free platform for the detection of NMP-22 based on CuAu nanowires decorated Co-MOFs (Figure 20B). The outstanding catalytic capabilities of Co-MOFs/CuAu NWs achieved a highly sensitive immunosensor with a good linear response (0.1 pg/mL–1 ng/mL), with a lower detection limit (33 fg/mL) suitable for the detection of NMP-22 from human urine samples. An immunoprobe based on AuNPs decorated Fe-MOF for the detection of PSA was reported by Feng et al. [150]. In this study, the labeling antibody was immobilized on AuNPs/Fe-MOF, and methylene blue (MB) covered by a thin layer of AuNPs-rGO served to covalently attach the coating antibodies. An amperometric signal at 0.18 V was measured to quantitatively measure PSA from urine samples (Figure 20C). Zhang et al. [27] reported a similar MB-based strategy for the detection of PSA (Figure 20D). Here, the MOF-325 adsorbed and stabilized MB, thereby solving the problem of MB leakage. A similar nanocomposite comprising MOF, rGO, and AuNPs was reported by Mehmandoust et al. [151] for the detection of a GFAP biomarker (Figure 20F). Here, AuNPs were anchored onto zeolitic imidazolate MOFs and were deployed as a recognition element for the detection of GFAP in urine samples. The intrinsic properties of unique nanomaterials are advantageous for specific immunosensor applications. Zhao et al. [152] fabricated an immunosensor for the detection of NMP-22 based on AuNPs and PtNPs decorated MOFs. The nanoparticles decorated MOF sowed an increased surface area to anchor antibodies through Pt-S and Au-N bonding (Figure 20E), and the immunosensor reported a sensitive response towards NMP-22. 

### 2.5. Biomarker Detection on Other 2D Materials Conjugated with Metal Nanoparticles

**Figure 21:**
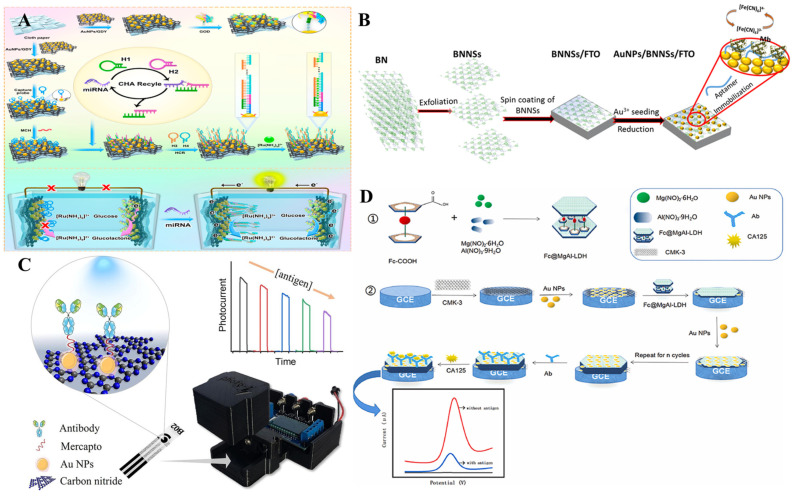
Schematic illustration of (**A**) Fabrication of a GDY-based self-powered device for miRNA-21 detection. Reprinted with permission from Ref. [164]. Copyright 2021 ACS, (**B**) Fabrication of AuNPs decorated boron nitride nanosheets based label-free aptasensor for the detection of the cardiac biomarker myoglobin. Reprinted with permission from Ref. [165]. Copyright 2019 Elsevier, (**C**) Graphitic carbon nitride sensitized with AuNPs for the PEC detection of CA15-3. Reprinted with permission from Ref. [170]. Copyright 2022 Elsevier, and (**D**) Fabrication of label-free electrochemical immunosensor based on LBL assembly of mesoporous carbon, AuNPs, and MgAl LDHs containing ferrocenecarboxylic acid. Reprinted with permission from Ref. [177]. Copyright 2022 Elsevier.

2D materials such as graphitic carbon nitride, black phosphorous, 2D layered double hydroxides (LDHs), boron nitrides, graphdiyne, etc. have also been explored in conjunction with metal nanoparticles for immunosensor applications with interesting biomarker targets (Figure 21, Table 5). Graphdiyne, the new 2D carbon allotrope with its unique sp-sp2 carbon network and highly π-conjugated structure has been receiving increased attention [163]. A graphdiyne-based self-powered biosensor platform was constructed by Hou et al. [164] for the determination of miRNA-21. Here, both the cathode and bioanode were fabricated by different modifications of AuNPs/GDY (Figure 21A). The 2D hexagonal boron nitride nanosheets, due to their electronic conductivity and large surface area were explored for immunosensor applications [165]. A label-free aptasensor for the detection of cardiac biomarker myoglobin on AuNPs decorated 2D-Boron nitride nanosheets was reported by Adeel et al. [166]. Here, the boron nitride nanosheets modified electrode AuNPs/BNNSs/FTO acted as a transducer for the immobilization of thiol-functionalized DNA aptamer for the specific binding of myoglobin (Figure 21B). Carbon nitrides are polymeric materials mainly consisting of carbon and nitrogen [167,168]. At ambient temperature, graphitic carbon nitride (g-C_3_N_4_) is the most stable allotrope of carbon nitrides. Due to the presence of basic surface groups and rich surface properties, g-C_3_N_4_ is attractive for many applications including catalysis [169]. Neto et al. [170] fabricated a miniaturized PEC system based on AuNPs decorated g-C_3_N_4_ for the detection of the breast cancer biomarker CA15-3 (Figure 21C). In this work, AuNPs on the g-C_3_N_4_ platform acted as a linker to 11-mercaptoundecanoic acid for the effective adsorption of antibodies. The performance of the PEC sensor was remarkable with a long linear range (0.1 fg/mL–10 ng/mL) and a very low detection limit (0.04 fg/mL). One of the promising candidates for immunosensor applications is 2D-Black phosphorus (BP) with high carrier mobility and controllable bandgap [171]. The unique properties of BP at atomic thickness are valuable for diverse applications [172,173,174]. Li et al. [175] reported a 2D-black phosphorous-supported Pt-Pd nanoelectrocatalyst for the determination of 4-AP, a potent biomarker for aniline exposure. Layered double hydroxides (LDHs) received attention because of their tunable chemistry and high charge density [176]. In one study, an electrochemical immunosensor based on AuNPs decorated ferrocene carboxylic acid conjugated MgAl layered double hydroxides for the label-free detection of CA-125 was reported by Wu et al. [177]. In this work, an LBL approach was used to increase the number of ferrocenes and antibodies, thereby amplifying the signal. The sensor reportedly displayed a wide linear range (0.01–1000 U/mL) and LOD (0.004 U/mL) and was tested for clinical cancer diagnostics (Figure 21D). 

## 3. Conclusions

In this review, we have discussed various electrochemical sensors that have been reported in recent years which incorporate various 2D nanomaterials conjugated with metal nanoparticles towards biomarker detection that have potential suitability for clinical use and some for point-of-care applications for cancer diagnosis. Although much research has been done in the synthesis of graphene, MoS_2_, MXenes, MOFs, and other 2D materials incorporated with metal nanoparticles for an in vitro analysis of biomarkers. However, significant progress needs to be done in performing an in vivo analysis. Moreover, due to their inherent conductivity, these 2D nanomaterials are significantly used in electrochemical or even optical sensing. However, they are often doped with other nanomaterials to improve their electroactivity/conductivity. Further, new approaches such as nanofabrication and clinical applicability are most crucial for developing an open-use-dispose type of sensor at low cost. Furthermore, electrode-to-electrode variations upon modifications with nanomaterials largely depend on the type of functionalization method adopted, which also needed to be studied for developing electrochemical transducers with greater stability and reproducibility. Finally, the paper-based electrochemical and wearable electrochemical sensing approaches for biomarker detections are also promising due to their improved sensitivity, selectivity, and portability, such as a simple paper-based sensor that can measure with an application able to get the electrochemical signal downloaded into a smartphone is best suitable for clinical/point-of-care applications [179,180]. Though the integration of microfluidic devices with electrochemical systems possesses numerous advantages, including rapid manipulation of sample fluid, reduced reagent consumption, and low cost, commercialization of these electrochemical sensors is still in its infancy due to the challenges that these techniques are facing, such as miniaturization (multiple electrodes and channels) and integration of microfluidic systems (miniaturized flow controllers). Therefore, it is necessary to develop manufacturable biosensors that can provide accurate quantification of a biomarker of interest with a meager quantity of solutions at point-of-care with simple fabrication steps by avoiding multiple modifications on the electrode surface.

## Figures and Tables

**Table 1:** Literature reports on the analytical parameters of graphene oxide conjugated nanoparticles for various biomarker detection.

Sensing Platform	Biomarker	Technique	Linear Range	LOD	Real Sample	Ref.
RGO-NP/ITO	CRP	EIS	1–10,000 ng/mL	0.08 ng/mL	Human serum	[36]
GO-CoPP	CPEB4	DPV	0.1 pg/mL–10 ng/mL	0.074 pg/mL	Human serum	[48]
AuNP-RGO/ITO	TNF-α	EIS	1–1000 pg/mL	0.43 pg/mL	Human serum	[49]
rGO@AgNPs	LA	CV	10–250 μM	0.726 μM	Human serum	[50]
AgPdNPs/rGO	RAC	LSA	0.01–100 ng/mL	1.52 pg/mL	----	[51]
	SAL			1.44 pg/mL		
	CLB			1.38 pg/mL		
MWCNTs-AuNPs/CS-AuNPs/rGO-AuNPs	OTC	DPV	1.00–540 nM	30 pM	----	[52]
GO-Fe_3_O_4_-β-CD	MGMT	DPV	0.001–1000 nM	0.0825 pM	Human plasma	[53]
AuNPs/GQDs/GO/SPCE	miRNA-21	SWV	0.001–1000 pM	0.04 fM	Human serum	[54]
	miRNA-155			0.33 fM		
	miRNA210			0.28 fM		
rGO/RhNPs/GE	HER-2-ECD	DPV	10–500 ng/mL	0.667 ng/mL	Human serum	[55]
AuNPs-rGO/ITO	IL8	DPV	500 fg/mL–4 ng/mL	72.73 pg/mL	----	[56]
Pd@Au@Pt/rGO	CEA	DPV	12 pg/mL–85 ng/mL	8 pg/mL	Human serum	[57]
	PSA		3 pg/mL–60 ng/mL	2 pg/mL		
AgNPs/GO/SPCE	PSA	DPV	0.75–100 ng/mL	0.27 ng/mL	Human serum	[58]
rGO-GNPs-Cr.6/GCE	L-Trp	SWV	0.1–2.5 μM	0.48 μM	Human serum	[59]
GO/AgNPs/Au	PSA	LSV	5–20,000 pg/mL	0.33 pg/mL	Human serum	[60]
AuNP/RGO/GCE	CA125	SWV	0.0001–300 U/mL	0.000042 U/mL	Human serum	[61]
ErGO-SWCNT/AuNPs	HER2	EIS	0.1 pg/mL–1 ng/mL	50 fg/mL	Human serum	[62]
Au-PtBNPs/CGO/FTO	MUC1	DPV	1 fM–100 nM	0.79 fM	Human serum	[63]
BNPAu-Fe-rGO/GCE	Acetaminophen	DPV	50–800 nM	0.14 nM	Human urine	[64]

**Table 2:** Literature reports on the analytical parameters of MoS_2_ conjugated nanoparticles for various biomarker detections.

Sensing Platform	Biomarker	Technique	Linear Range	LOD	Real Sample	Ref.
Au-NPs/MoS_2_	CRP	EIS	1 fg/mL–1 µg/mL	0.01 fg/mL	----	[83]
Fe_3_O_4_@MoS_2_-AuNPs	H_2_O_2_	SWV	1–120 μM	80 nM	Human serum	[84]
Au/MoS_2_/Au/PET	GP120	SWV	0.1 pg/mL–10 ng/mL	0.066 pg/mL	Human serum	[85]
MoS_2_/Pt@Au-nanoprism/PDA	free-PSA; total-PSA	DPV	0.0001–100 ng/mL	0.1 pg/mL; 0.0011 fg/mL	Human serum	[86]
MoS_2_ NFs/Au@AgPt YNCs	CEA	i-t curve	10 fg/mL–100 ng/mL	3.09 fg/mL	Human serum	[87]
Au/Co-BDC^f^/MoS_2_	CTnI^g^	i-t curve	10 fg/mL–100 ng/mL	3.02 fg/mL	Human serum	[88]
Au/MoS_2_/rGO	CA 27-29 BCA	i-t curve	0.1–100 U/mL	0.08 U/mL	Human serum	[89]
MoS_2_-AnNPs/GCE	CEA	DPV	1 pg/mL–50 ng/mL	0.27 pg/mL	Human serum	[90]
Ce-MoS_2_/AgNRs	PSA	CV	0.1–1000 ng/mL	0.051 ng/mL	Human serum	[91]
MoS_2_@Au	Siglec-5	ECL	10 pM–500 pM	8.9 pM	Human serum	[92]
MoS_2_/PPY/AuNPs	Glucose	DPV	0.1–80 nM	0.08 nM	Human serum	[93]
AgPt/MoS_2_	H_2_O_2_	i-t curve	20 μM–4 mM	1.0 μM	----	[94]

**Table 3:** Recent literature reports on metal nanoparticles incorporated MXenes for electrochemical biomarker detection.

Sensing Platform	Biomarker	Technique	Linear Range	LOD	Real Sample	Ref.
AuNPs/Ti_3_C_2_@PAMAM	cTnT	DPV	0.1–1000 ng/mL	0.069 ng/mL	Human serum	[108]
Ti_3_C_2_@AuNPs	PSA	DPV	pg/mL	3.0 fg/mL	Plasma	[109]
L-cys/AuNP/Ti_3_C_2_	Cortisol	CA	5–40 ng/mL	0.54 ng/mL	Artificial sweat	[110]
PdPtBP MNPs/Ti_3_C_2_	KIM-1	DPV	0.5–100 ng/mL	86 pg/mL	Human urine	[112]
AuNPs-Ti_3_C_2_/AuE	miRNA-21	DPV	100 aM–1 nM	50 aM	----	[113]
AuNP@MXene/Au	miRNA-21	DPV	500 aM–50 nM	204 aM	Total plasma	[114]
	miRNA-141			138 aM		
cDNA-Fc/MXene/Apt/Au/GCE	MUC1	SWV	0.001–1.0 × 10^4^ nM	0.33 × 10^−3^ nM	Human serum	[115]
AuNP-Ti_3_C_2_	CYFRA21-1	SWV	0.5–1.0 × 10^4^ pg/mL	0.1 pg/mL	Human serum	[116]
MCH/CP/MXene-Au/GCE	miRNA-377	SWV	10 aM–100 pM	1.35 aM	Human serum	[118]
Ti_3_C_2_-AuNPs/GCE	PSA	DPV	1–50,000 pg/mL	0.31 pg/mL	----	[119]
AuNPs/d-S-Ti_3_C_2_	PCT	DPV	0.01–1.0	2.0 fg/mL	----	[120]
MB/DNA/HT/HP1/AuNPs/Ti_3_C_2_/BiVO_4_/GCE	VEGF_165_	PEC	10 fM–100 nM	3.3 fM	----	[121]

**Table 4:** Recent literature reports on metal nanoparticles incorporated MOFs for electrochemical biomarker detection.

Sensing Platform	Biomarker	Technique	Linear Range	LOD	Real Sample	Ref.
Au/MOF-235/MB	PSA	DPV	0.01–1.2 ng/mL	3 pg/mL	Human serum	[28]
Co-MOFs/CuAu NWs	NMP-22	CA	10^−4^–1 ng/mL	33 fg/mL	Human urine	[149]
AuNPs/Fe-MOF	PSA	SWV	0.001–100 ng/mL	0.13 pg/mL	Human serum	[150]
Au@ZIF-8@rGO/SPE	GFAP	EIS	50–10,000 fg/mL	50 fg/mL	Human urine	[151]
rGO-TEPA/AuNPs-PtNPs-MOFs	NMP-22	DPV	0.005–20 ng/mL	1.7 pg/mL	Human urine	[152]
PtNPs/Fe-MOF	Thrombin	DPV	1 fM–10 nM	0.33 fM	Human serum	[156]
Fe_3_O_4_@UiO-66/Cu@Au	cTnI	DPV	0.05–100 ng/mL	16 pg/mL	Human serum	[157]
SiO_2_-Fc-COOH-Au/UiO-66-TB	PCT	DPV	1 pg/mL–100 ng/mL	0.3 pg/mL	Human serum	[158]
Au-MoS_2_/MOF	NSE	CA	1 pg/mL–100 ng/mL	0.37 pg/mL	Human serum	[159]
AgNPs@Co/Ni-MOF	AFP	ECL	1 pg/mL–100 ng/mL	0.417 pg/mL	Human serum	[160]
BSA/Ab-AgNPs/CdS@MOF-5/PDDA/FTO	cTnI	ECL	0.01–1000 pg/mL	5.01 fg/mL	Human serum	[161]
Pd/NH_2_-ZIF-67	PSA	CA	100 fg/mL–50 ng/mL	0.03 pg/mL	Human serum	[162]

**Table 5:** Recent literature reports on biomarker detection based on various metal nanoparticles decorated 2D materials.

Sensing Platform	Biomarker	Technique	Linear Range	LOD	Real Sample	Ref.
AuNPs/GDY	miRNA-21	OCV	0.1–100,000 fM	0.034 fM	Human serum	[164]
Au-NPs/2D-hBN/FTO	Mb	DPV	0.1–100 μg/mL	34.6 ng/mL	Human serum	[166]
AuNPs-g-C_3_N_4_	CA15-3	PEC	10^−7^–10^1^ ng/mL	0.04 fg/mL	Human serum	[170]
Pt-Pd/BP	4-AP	DPV	0.02–5 μM	14.1 nM	----	[175]
Au/Fc@MgAl-LDH	CA-125	DPV	0.01 U/mL–1000 U/mL	0.004 U/mL	Human serum	[177]
AuNRs-g-C_3_N_4_	NS1	EIS	0.6–216 ng/mL	0.09 ng/mL	Human serum	[178]

## Data Availability

Not applicable.

## Abbreviations

2D	Two dimensional
2D-Hbn	2D-hexagonal boron nitride
AgNPs	Silver nanoparticles
AgNRs	Silver nanorods
Apt	Aptamer
Au	Gold
AuE	Gold electrode
AuNP-RGO	Au nanoparticle-reduced graphene oxide
AuNPs	Gold nanoparticles
AuPtBNPs	Gold platinum bimetallic nanoparticles
BDC	1,4-benzenedicarboxylate
BiVO_4_	Bismuth vanadate
BNNSs	Boron nitride nanosheets
BP	Black phosphorous
BSA	Bovine serum albumin
CA 27-29 BCA	Cancer antigen 27-29 breast cancer antigen
CA	Chronoamperometry
CA125	Cancer antigen 125
CA15-3	Cancer antigen 15-3
C-DNA	Capture DNA
CEA	Carcinoembryonic antigen
ce-MoS_2_	Chemical exfoliated MoS_2_
CGO	Carboxylic groups
CLB	Clenbuterol
CoPP	Cobalt protoporphyrin
CP	Capture probe
CPEB4	Cytoplasmic polyadenylate element-binding protein 4
Cr.6	18-crown-6
CRP	C-reactive protein
CS	Chitosan
CTnI	Cardiac troponin I
CTnT	Cardiac troponin T
CV	Cyclic voltammetry
CYFRA21-1	Cytokeratin 19 fragment
DNA	Deoxyribonucleic acid
DPV	Differential pulse voltammetry
ECD	Extracellular domain
ECL	Electrochemiluminescence
EIS	Electrochemical impedance spectroscopy
ELISA	Enzyme-linked immunosorbent assay
eT	Electron transfer
Fc	Ferrocene
FTO	Fluorine doped tin oxide
g-C_3_N_4_	Graphitic carbon nitride
GCE	Glassy carbon electrode
GDY	Graphdiyne
GE	Graphite electrode
GFAP	Glial fibrillary acidic protein
GP120	Glycoprotein GP120
4-AP	p-Aminophenol
HER-2	Human epidermal growth factor receptor-2
HP1	Hairpin DNA
HT	Hexane thiol
IL8	Interleukin-8
i-t curve	Amperometric current-time response
ITO	Indium tin oxide
LA	Lactic acid
LBL	Layer by layer
L-cys	L-Cysteine
LOD	Limit of detection
LSV	Linear sweep voltammetry
L-Trp	L-tryptophan
Mb	Myoglobin
MCH	6-mercaptohexanol
MgAl-LDH	Mg-Al-Layered double hydroxide
MGMT	O6-methylguanine-DNA methyltransferase
miRNA-141	micro-RNA-141
miRNA-21	micro-RNA-21
miRNA-377	micro-RNA-377
miRNAs	micro-RNAs
MNPs	Mesoporous nanoparticles
MOFs	Metal organic frameworks
MUC1	Mucin1
MWCNT	Multiwalled carbon nanotubes
NMP-22	Nuclear matrix protein 22
NS1	Non-structural 1
NSE	Neuron-specific enolase
OCV	Open circuit voltage
OTC	Oxytetracycline
PAMAM	Polyamidoamine
PCT	Procalcitonin
PDA	Polydopamine
PdPtBP MNPs	Pd-Pt-Black phosphorous-mesoporous nanoparticles
PEC	Photoelectrochemical
PET	Polyethylene terephthalate
PPY	Polypyrrole
PSA	Prostate specific antigen
PtNPs	Platinum nanoparticles
RAC	Ractopamine
rGO	Reduced graphene oxide
RhNPs	Rhodium nanoparticles
RNA	Ribonucleic acid
S/N	Signal-to-noise ratio
SAL	Salbutamol
SPCE	Screen-printed carbon electrode
SWV	Square wave voltammetry
TEPA	Tetraethylenepentamine
VEGF165	Vascular endothelial growth factor 165
YNCs	Yolk-shell nanocubes
β-CD	β-cyclodextrin
